# Automatic Detection of Abnormalities and Grading of Diabetic Retinopathy in 6-Field Retinal Images: Integration of Segmentation Into Classification

**DOI:** 10.1167/tvst.11.6.19

**Published:** 2022-06-22

**Authors:** Jakob K. H. Andersen, Martin S. Hubel, Malin L. Rasmussen, Jakob Grauslund, Thiusius R. Savarimuthu

**Affiliations:** 1 The Maersk Mc-Kinney Moeller Institute, SDU Robotics, University of Southern Denmark, Odense, Denmark; 2 Department of Ophthalmology, Odense University Hospital, Odense, Denmark; 3 Department of Clinical Research, University of Southern Denmark, Odense, Denmark; 4 Steno Diabetes Center Odense, Odense University Hospital, Odense, Denmark

**Keywords:** deep learning, lesion detection, diabetic retinopathy, decision support, automatic diagnosis

## Abstract

**Purpose:**

Classification of diabetic retinopathy (DR) is traditionally based on severity grading, given by the most advanced lesion, but potentially leaving out relevant information for risk stratification. In this study, we aimed to develop a deep learning model able to individually segment seven different DR-lesions, in order to test if this would improve a subsequently developed classification model.

**Methods:**

First, manual segmentation of 34,075 different DR-lesions was used to construct a segmentation model, with performance subsequently compared to another retinal specialist. Second, we constructed a 5-step classification model using a data set of 31,325 expert-annotated retinal 6-field images and evaluated if performance was improved with the integration of presegmentation given by the segmentation model.

**Results:**

The segmentation model had higher average sensitivity across all abnormalities compared to the retinal expert (0.68 and 0.62) at a comparable average F1-score (0.60 and 0.62). Model sensitivity for microaneurysms, retinal hemorrhages and intraretinal microvascular abnormalities was higher by 42.5%, 8.8%, and 67.5% and F1-scores by 15.8%, 6.5%, and 12.5%, respectively. When presegmentation was included, grading performance increased by 29.7%, 6.0%, and 4.5% for average per class accuracy, quadratic weighted kappa, and multiclass macro area under the curve, with values of 70.4%, 0.90, and 0.92, respectively.

**Conclusions:**

The segmentation model matched an expert in detecting retinal abnormalities, and presegmentation substantially improved accuracy of the automated classification model.

**Translational Relevance:**

Presegmentation may yield more accurate automated DR grading models and increase interpretability and trust in model decisions.

## Introduction

Diabetic retinopathy (DR) is the most frequent complication in diabetes,^1^ which is the most common metabolic disease in the working aged population of the Western world.^2^ Regular DR screening has been proven to reduce incidence of severe DR related vision loss by 90%.^3^ In clinical practice, retinal experts will often grade the severity of DR based on some predefined criteria using a disease severity scale. The International Clinical Diabetic Retinopathy (ICDR) disease severity scale^4^ is a widely adopted standard for DR disease classification. The standard proposes a five-point scale with levels ranging from no DR (level 0) to proliferative DR (PDR, level 4) with three intermediate levels of increasing severity; mild nonproliferative DR (mild NPDR, level 1), moderate nonproliferative DR (moderate NPDR, level 2), and severe nonproliferative DR (severe NPDR, level 3). The lowest level of disease (level 1) is indicated by microaneurysms (MA). Level 2 is defined as the presence of more than MA, or hemorrhages (HEM) but less severe than level 3, where definite venous beadings or prominent intraretinal microvascular abnormalities (IRMA) are also defining features. Level 4 is indicated by neovascularizations (NV; both active or treated by panretinal photocoagulation) or vitreous hemorrhages. If left untreated, DR level 4 may result in irreversible vision loss. Other lesions often present in the retina of people with DR are hard exudates (HE) and cotton wool spots (CWS).

At present, DR grading is most commonly a manual task, but, in recent years, the interest in automating this task has increased significantly due to the performance of deep learning models and convolutional neural networks (CNNs) for different image recognition tasks, such as image classification, segmentation, and object detection.

Gulshan et al.^5^ were among the first to demonstrate the use of a CNN for automatic detection of referable DR, defined as moderate NPDR or higher on the ICDR scale, or diabetic macular edema. Their CNN was trained on over 100,000 single field retinal images with reference grades assigned by multiple retinal experts to perform image classification of the aforementioned disease levels. Many similar works on the use of classification CNNs also use a binary standard for classification, either no or mild NPDR versus referable DR (moderate NPDR or worse) or no DR versus all other ICDR levels.^6^^–^^10^

Ideally, computer assisted grading systems should be able to recognize all levels of DR, as this would increase the applicability of the systems in real world clinical practice. In order to do so, the underlying model (for example neural network), has to be able to recognize the features (abnormalities) used by human experts when grading images according to, for example, the ICDR reference standard.

Studies on deep learning for disease staging across multiple levels have been performed.^11^^–^^13^ Results of these studies indicate that for certain levels of disease, the specific features, such as MA, IRMA, or NV, are difficult for models to detect, resulting in somewhat disparate levels of accuracy for individual levels. This is likely caused by the need to reduce image resolution prior to development of deep learning models in order to overcome computational memory constraints imposed by the graphical processing unit on which CNNs are developed. As discussed by Krause et al.,^13^ this leads to reduction in the effective feature resolution, which makes it challenging for networks to detect these abnormalities. When multiple fields or wide field retinal images with larger resolutions are used, this reduction in feature resolution becomes even more pronounced.

Although CNNs are approaching human level performance for automatic detection and grading of DR, they are still lacking in interpretability. The conclusions reached by deep learning algorithms are in most cases opaque, which may serve as a barrier for adoption into real world clinical practice. This problem could be alleviated by utilizing networks specifically trained to detect the abnormalities used by human experts in the grading process. Retinal abnormalities are often small or their intrinsic features make them hard to accurately discern. Furthermore, accumulating the data needed to train algorithms capable of this task is challenging, as it not only requires collecting images, but also the difficult and demanding task of hand annotating each individual relevant pixel in them.

Automatic medical image segmentation has, like classification, been greatly improved by the advancement in deep learning methods.^14^ Several works have been dedicated to segmentation of MA,^15^^–^^19^ HEM,^19^^–^^21^ HE, and CWS,^19^^,^^22^^–^^24^ and retinal vessels.^25^^–^^27^ Segmentation of IRMA, panretinal photocoagulation scars (PC) and NV in retinal fundus images have at present, to the best of our knowledge, not been explored to the same degree. Models that are able to accurately detect these specific abnormalities could serve as an important part of an automatic grading method, as a way to minimize the adverse effect of reducing input resolution, and in turn improve automatic grading models’ ability to stratify DR across all levels.

In the studies on grading DR or segmentation of retinal abnormalities referenced above, single-field images, or in one case two-field retinal images^12^ are used. A lot of the features and abnormalities relevant for grading DR may be located outside this field of view. As such, the additional information obtained by including multiple fields or using wide field images should lead to more accurate diagnosis.

The data sets in this study consisted of six-field retinal images from Danish patients of the type used in the Danish DR screening program. This work is the first to investigate the use of deep learning models in this population, and, to the best of our knowledge, the first to perform segmentation of retinal abnormalities and grading of DR in six-field retinal images.

## Methods

Two models were developed in this study; a segmentation model for segmentation and detection of retinal abnormalities and a classification model for automatic grading of DR disease severity into the five levels on the ICDR scale.

The data set used for developing the segmentation model consisted of 300, high resolution, 6,528 × 6,528 pixel six-field retinal fundus images obtained from hospitals in the Region of Southern Denmark. Each image contained pixel level annotations for presence of the following retinal abnormalities found in the retina of people with suspected DR: MA, HEM, CWS, HE, IRMA, NV, and PC. Images had been annotated by two retinal experts independent from each other using a proprietary data annotation tool.^28^ The quality of the data set was validated using the intraclass correlation coefficient (ICC) as a measure of agreement between experts for each abnormality type. Good to excellent agreement was found for MA (0.81), HEM (0.83), HE (0.91), CWS (0.91), IRMA (0.77), and PC (0.99). NV was the only abnormality for which low agreement was found, with an ICC value of 0.07. Details on validation of the data are described in Grauslund et al.^29^

**Figure 1.**
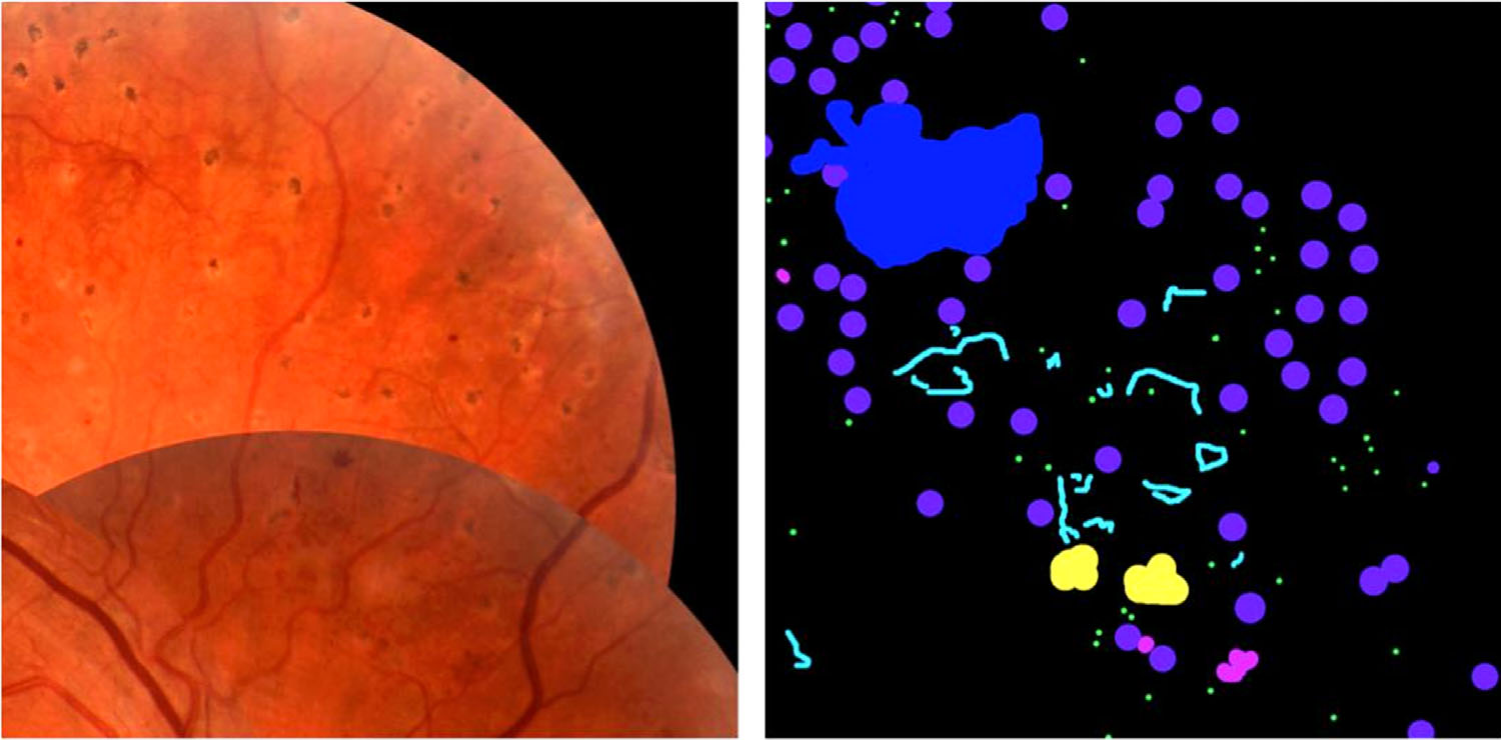
Example feature image crop (*left*) and expert annotations (*right*) with annotations for MA (*green*), HEM (*magenta*), CWS (*yellow*), IRMA (*cyan*), NV (*blue*), and PC (*purple*).

The data set was split into training, tuning, and testing sets of 209, 45, and 46 images, respectively. For training and evaluation of model performance, one set of expert annotations was used and the annotations from the second expert were used for comparative analysis. Images were split such that approximately 70% of images containing NV were assigned to the training set and approximately 30% divided between tuning and test sets. Remaining images were also divided using the same 70%, 15%, and 15% split. Figure 1 shows an example image with different abnormalities along with retinal expert pixel level annotations.

The images from the first expert annotator in the training, tuning, and test sets contained 34,075 pixel-level annotations for the abnormality types described above. Table 1 gives a detailed overview of the number of annotations in the three data set splits

**Table 1.** Number of Annotations for each Abnormality From the First Expert Across Data Set Splits

	Abnormality							
Split	MA	HEM	PC	HE	CWS	IRMA	NV	Total
Training	11,024	2,622	7,148	2,357	385	846	157	24,539
Tuning	1,840	552	1,535	338	76	220	23	4,584
Testing	2,018	452	1,676	348	93	335	30	4,952
Total	14,882	3,626	10,359	3,043	554	1,401	210	34,075

The top row gives the abbreviation for each abnormality type in the data set and the rightmost column and bottom row holds the total number of abnormalities in each of the splits as well as the full data set.

To improve the stability of the trained network, the optic nerve was annotated by hand prior to training so that the network learned this feature as well. In cases where the expert had made markings on the optic nerve, the expert annotation took precedence.

The model used was a variation of the U-net encoder-decoder architecture^14^ equipped with a Inception-v3 encoder^30^ pretrained on ImageNet.^31^ The final pixel-wise classification layer of the decoder was modified to consist of three different K × K × N kernels with K = 1, 3, and 5, respectively, and N = to the number of classes (abnormalities and background features). During training, the loss calculated from each filter's prediction was assigned an equal weight of 0.33. The final pixel-wise class prediction from the trained model was based on the maximum average softmax probability from the three output kernels. The model was implemented in Keras^32^ and pretrained ImageNet weights were obtained through its applications module. The network was trained on 256 × 256 × 3 pixel patch sampled from the 209 high resolution training images using a sliding window approach. Random data augmentations, such as vertical and horizontal flipping and shifting, as well as gamma adjustment were performed. Inference on the full resolution 6,528 × 6,528 images was performed using an overlapping tiles strategy by modifying the input layer of the model to accept 1,024 × 1,024 resolution image crops and combining the tiles into a full resolution segmentation mask by cropping the edge pixels of the tiles to remove segmentation errors in the boundary regions.

Using the first expert as reference standard, the segmentation model and the second expert were evaluated on the ability to detect individual abnormalities as well as identify images containing one or more of a specific type of abnormality. Performance was measured using recall and precision metrics as well as F1 scores for both tasks. Recall is equivalent to sensitivity (that is, the number of true positives divided by the sum of true positives and false negatives). Precision is equivalent to the positive predictive value, which is the number of true positive predictions divided by the sum of true positives and false positives. The F1 score is the harmonic mean of recall and precision defined as two times the product of precision and recall, divided by the sum of the two metrics.

For detection of individual abnormalities in the 46 test images, a true positive was counted if one or more pixels in individual abnormalities of a specific type predicted by the model or second expert overlapped with one or more pixels of an abnormality of the same type in the reference annotation. If abnormality pixels did not overlap, the prediction was counted as a false positive. Conversely, if one or more pixels of individual abnormalities in the reference annotation did not overlap with abnormalities of the same type in the prediction masks from the model and second expert, they were counted as false negatives.

For identifying images containing a specific type of abnormality, a true positive was counted if one or more abnormalities of a specific type was predicted in images where the reference annotation also contained at least one abnormality of the same type. Otherwise, a false positive was counted. A false negative was counted if the model or second expert failed to identify any abnormalities of a specific type in an image where this type had been annotated by the reference.

For development of the classification model, a data set of 31,325, 6-field high resolution images was used. Images were obtained from 5,127 patients screened at Odense University Hospital, a hospital located on the island of Fyn in the Region of Southern Denmark, Denmark. The mean age of patients was 54.7 years (±15.6), and the average number of screening episodes was 3.1 (±1.9). Overall, 40.0% of patients had type 1 diabetes and 53.3% had type 2 diabetes. The remaining 6.7% had either other or unknown diabetes type. The disease severity grade for each image was given by a retinal expert and assigned based on patient records from Fyens Diabetes Database. The data set was split into training, tuning, and test sets using a 75%, 10%, and 15% split. The data set was split such that images from individual patients only appeared in one of the three subsets. A detailed overview of the distribution of image grades in each split is given in Table 2.

**Table 2.** Number of Images for each Level on the ICDR Scale in each Subset of the Development Data Set for the Classification Model

	Level 0	Level 1	Level 2	Level 3	Level 4	Total
Training (75%)	11,926	2,863	4,797	1,490	2,367	23,443
Tuning (10%)	1,595	369	632	191	392	3,179
Testing (15%)	2,361	575	797	310	660	4,703
Total	15,882	3,807	6,226	1,991	3,419	31,325

**Figure 2.**
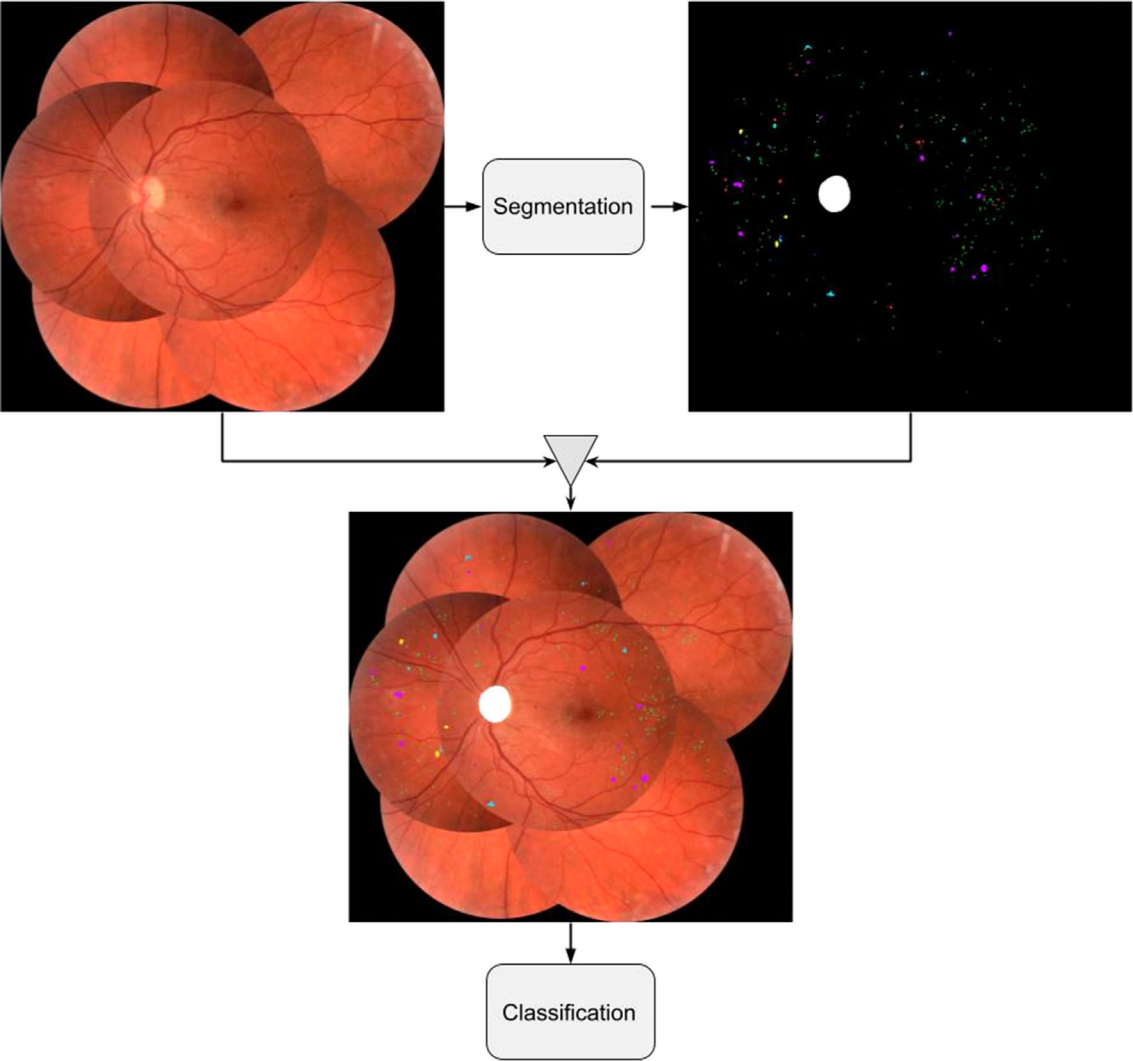
Preprocessing step consisting of superimposing segmentation model outputs on development images for the classification model.

The classification model was an Inception v3 network similar to that used by Gulshan et al.^5^ for automatic detection of referable DR as well as Sahlsten et al.^12^ and Krause et al.^13^ for automatic grading of all DR levels. In this study, two classification models were developed and their performance with regard to full scale grading of disease was compared. One model was developed on raw image features, meaning that no preprocessing apart from standardization and normalization was applied to the images prior to training. The second model was developed on images where presegmentation of abnormalities had been performed using the segmentation model. In this process, the segmentation model constructed a segmentation mask with predicted abnormalities for each image in the data set. To allow the model to consider both the raw image features as well as the presegmented abnormalities, the segmentation mask was superimposed on the raw feature image prior to training. This process is illustrated in Figure 2.

Similar to Sahlsten et al.^12^ and Krause et al.,^13^ the input size to the network was changed. Resolution was increased from the original 299 × 299 pixels to 598 × 598 pixels. To further reduce the adverse effect of downsampling, the black border around the retina was cropped from the images.

## Results

### Segmentation

**Figure 3.**
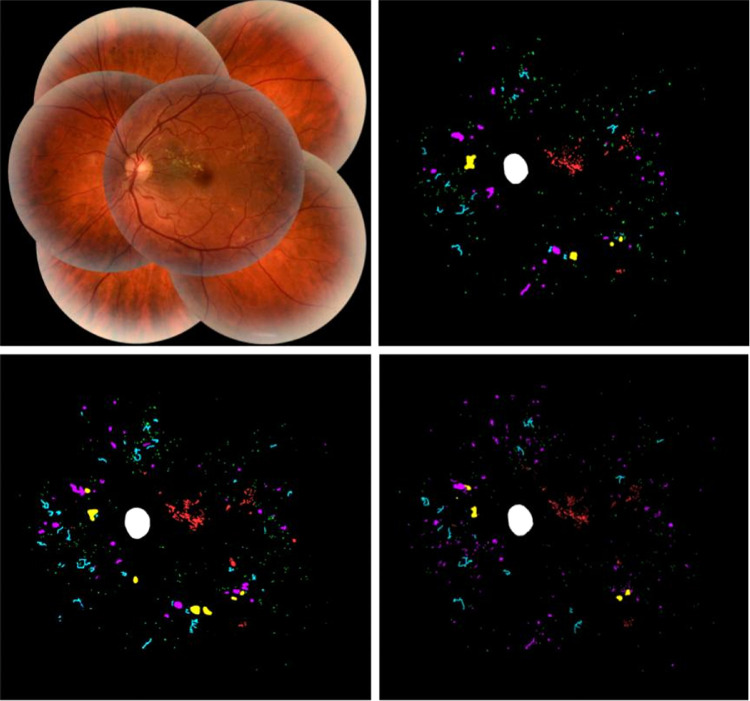
Example feature image (*top left*), reference annotation (*top right*), network segmentation output by the segmentation model (*bottom left*), and annotations from the second expert (*bottom right*). MA (*green*), HEM (*magenta*), IRMA (*cyan*), HE (*red*), and CWS (*yellow*).

Output masks from the segmentation model and annotations from the second expert along with the reference annotation and feature image from the test set of the segmentation data set are shown in Figure 3.

Results for the trained model as well as those of the second expert for detecting individual abnormalities using precision and recall detection metrics and F1 scores with expert 1 as reference are given in Table 3.

**Table 3.** Precision, Recall, and F1 Score for the Model and Second Expert Using Expert 1 as Reference

		Expert 1 Reference							
		MA	HEM	CWS	HE	PC	IRMA	NV	Mean
Model	Precision	0.66	**0.59**	0.38	0.63	0.82	0.41	0.40	0.52
	Recall	**0.67**	**0.74**	**0.46**	0.61	0.84	**0.67**	0.77	**0.68**
	F1	**0.66**	**0.66**	0.42	0.62	0.83	**0.51**	0.53	0.60
Expert 2	Precision	**0.73**	0.57	**0.75**	**0.70**	**0.90**	**0.54**	**0.45**	**0.66**
	Recall	0.47	0.68	0.41	**0.71**	**0.85**	0.40	**0.88**	0.62
	F1	0.57	0.62	**0.53**	**0.70**	**0.87**	0.46	**0.60**	**0.62**

The top row gives the abbreviation of each abnormality type. Results are compared column-wise and the bold face number in each column indicates the highest metric value between expert and model for specific abnormalities as well as the mean metric value across all abnormalities. Rows are shaded to improve readability.

Table 4 gives precision and recall values and F1 scores for detecting images containing specific abnormalities in the test set for the model compared to the second expert using expert 1 as reference.

**Table 4.** Image Level Precision, Recall, and F1 Score as well as the Mean Metric Value Across all Abnormalities in the Segmentation Data Test Set for Expert 2 and Model

		Expert 1 Reference							
		MA	HEM	CWS	HE	PC	IRMA	NV	Mean
Model	Precision	0.81	0.78	0.50	0.53	0.27	0.69	0.35	0.56
	Recall	**0.97**	**1.00**	**0.92**	**1.00**	**1.00**	**1.00**	0.86	**0.96**
	F1	0.88	0.88	0.65	0.69	0.43	0.82	0.50	0.69
Expert 2	Precision	**0.97**	**0.95**	**1.00**	**0.85**	**1.00**	**1.00**	**0.70**	**0.92**
	Recall	0.86	0.86	**0.92**	0.68	**1.00**	**1.00**	**1.00**	0.90
	F1	**0.91**	**0.90**	**0.96**	**0.76**	1.00	**1.00**	**0.82**	**0.91**

For image level, a true positive was counted if a type of abnormality was detected in an image containing any such abnormality according to the reference.

Table 5 shows how the network and expert 2 confused similar looking abnormalities or incorrectly detected background (BG) as abnormal.

**Table 5.** Confusion Table Showing False Positive Detected Abnormalities Confused With Other Structures in the Segmentation Data Test Set for the Model and Expert 2


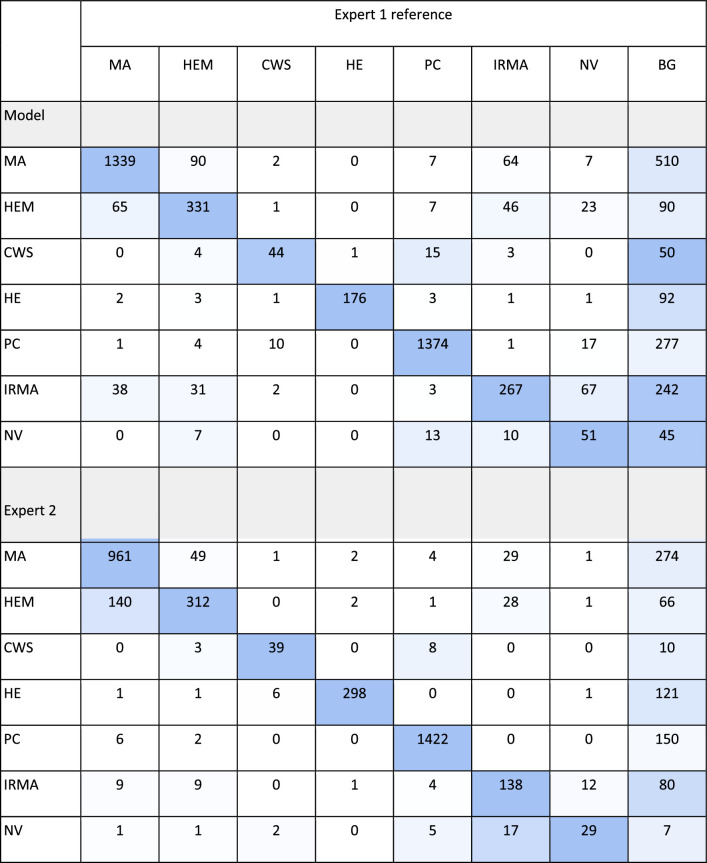

The diagonally colored cells indicate the true positive detections. The color gradient symbolizes the number of confused abnormalities or background features (BG) relative to the number of true positive detections of each abnormality type.

**Figure 4.**
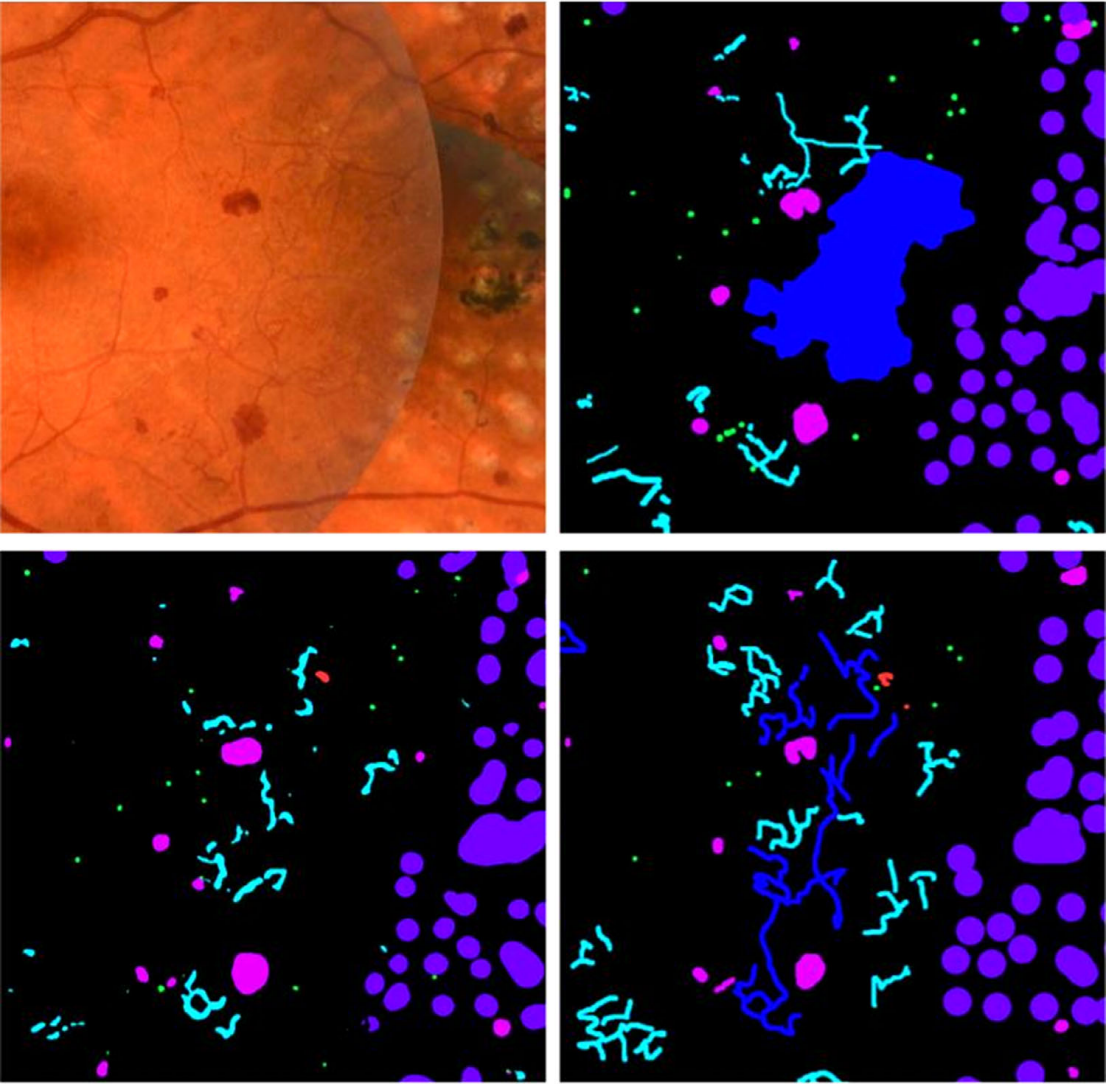
Examples of abnormalities confused by the model and second expert. Feature image crop (*top left*), reference annotation (*top right*), model output (*bottom left*), and expert 2 annotations (*bottom right*). MA (*green*), HEM (*magenta*), IRMA (*cyan*), HE (*red*), PC (*purple*), and NV (*blue*).

The example in Figure 4 illustrates how abnormalities with similar characteristics were sometimes confused by the model as well as expert 2. In the example shown, the model incorrectly detected IRMA changes in a region where the reference annotation was given as NV. The second expert also identified some abnormalities in the same area as being IRMA changes, although many of the detections were correctly given as NV according to the reference. The example also shows instances of MA, HEM, and IRMA being confused. The supplementary material gives adjusted precision numbers for detection of individual abnormalities. That is, abnormalities were counted as true positives regardless of the type of abnormalities they overlapped in the reference annotation.

**Figure 5.**
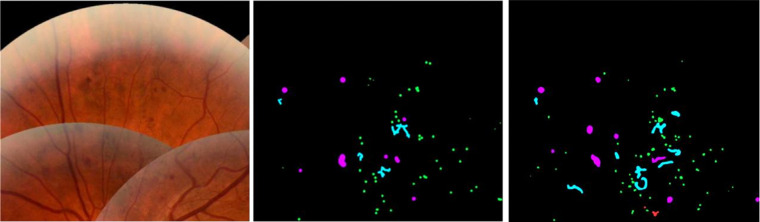
Example showing feature image region (*left*) where corresponding reference annotation (*middle*) and segmentation model predictions (*right*) are somewhat mismatched but still mostly contain the same types of abnormalities. MA (*green*), HEM (*magenta*), IRMA (*cyan*), and HE (*red*).

Sometimes, the segmentation model detected specific abnormalities while overlooking others in regions where these had been annotated by the reference annotator. Meaning that even though the model failed to detect individual lesions, it did identify regions of interest where these were present. In the same way, background features were identified as abnormal in regions where other pixels had been annotated by the reference. This may be realized by looking at the example in Figure 5.

Table 6 shows the number of falsely positive detected images containing either some or no abnormalities by the model and expert 2. The tables should be interpreted such that for NV, 6 images were accurately detected by the model and a total of 11 images were wrongly predicted to contain NV. Of these, 11 images contained MA, 10 contained MA and CWS, 9 contained MA, CWS, HE, and HEM, 8 images contained IRMA, HE, CWS, HEM, and MA, and 5 contained all abnormalities apart from NV: IRMA, HE, CWS, HEM, MA, and PC. In the five images where the model predicted MA but that did not contain abnormalities according to the reference, two MA on average were incorrectly detected.

**Table 6.** Confusion Table Showing False Positive Detected Abnormal Images Containing Either Other or no Abnormalities for the Model and Expert 2


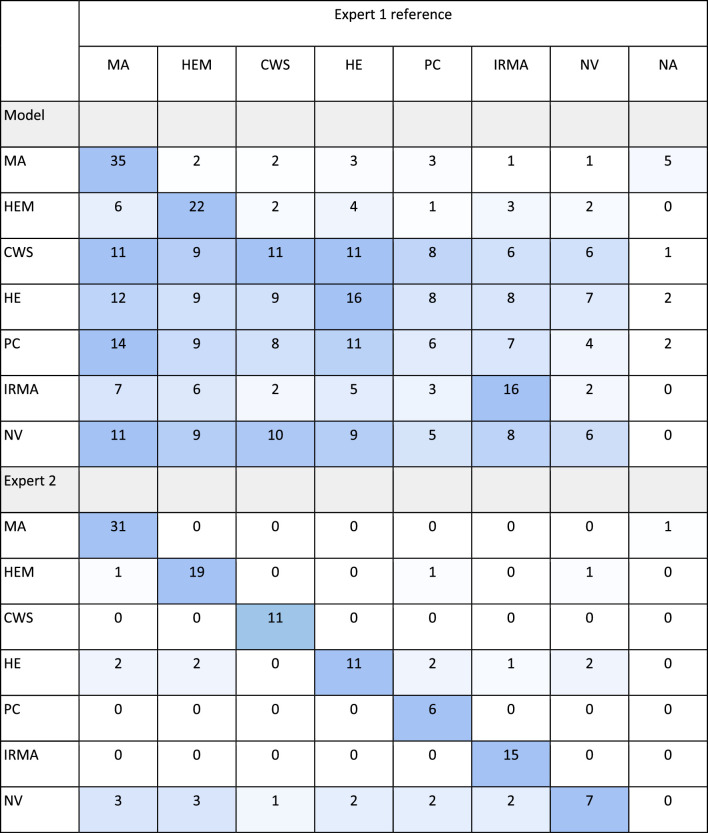

Diagonally shaded cells indicate true positive detected images, that is, images containing one or more of the specific abnormality type detected by the network and expert 2 using expert 1 as reference. The color gradient symbolizes the number of incorrect detections relative to the number of true positive detections for each abnormality type or images with no abnormalities (NA).

### Grading

**Figure 6.**
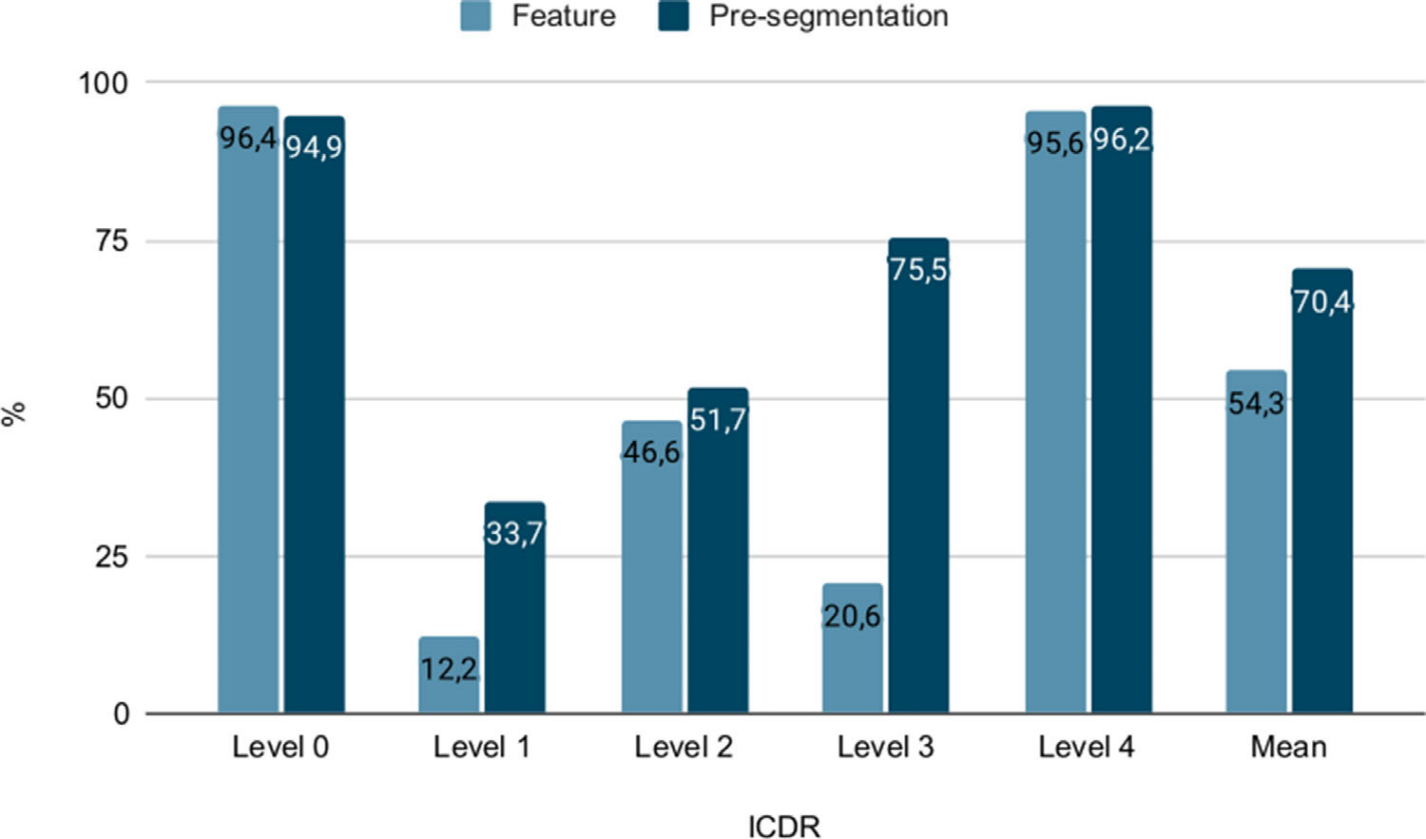
Compared per class accuracy for individual disease levels on the ICDR scale for model trained on raw image features and model trained with presegmented abnormalities.

Per class grading accuracy for the two classification models trained with and without presegmented abnormalities are compared in Figure 6. The average per class accuracy was 54.3% for the model trained only on the raw image features and 70.4% for the model trained on images with presegmented abnormalities.

**Figure 7.**
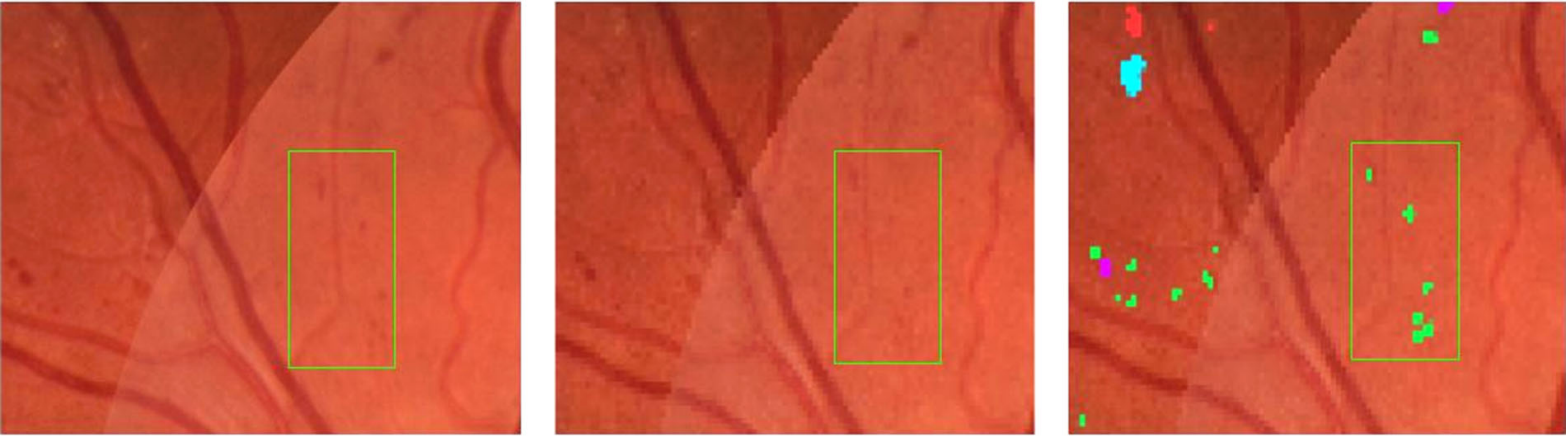
Comparison between resolution of microaneurysms in a region cropped from an original resolution image (*left*) and the same region from a downsampled feature image (*middle*) and downsampled presegmented image (*right*). MA (*green*), HEM (*magenta*), IRMA (*cyan*), and HE (*red*).

The most noticeable differences in accuracy between the two models was with level 1 DR and level 3 DR, with improved accuracy of 21.5 percentage points and 54.9 percentage points, respectively. These levels are indicated by microvascular abnormalities, such as MA in the case of level 1 and IRMA in the case of level 3. Figure 7 illustrates the difference in downscaling images with regard to the resolution of relevant microvascular image features, such a MA.

Confusion tables for full scale grading of DR for the two classification models are shown in Table 7. From the confusion tables, the quadratically weighted kappa was calculated as a measure of agreement between the models and the reference gradings. The model trained on images without presegmented abnormalities had a quadratically weighted kappa value of 0.85, and for the model trained on images with presegmented abnormalities the value was 0.90. The multiclass macro average area under the curve for the model developed on only raw image features was 0.88, whereas it was 0.92 when presegmentation of abnormalities was used.

**Table 7.** Confusion Table for Full Scale Grading of DR According to the ICDR Scale in Six-Field Images for the Feature Image Model and Model Trained Using Presegmented Abnormalities


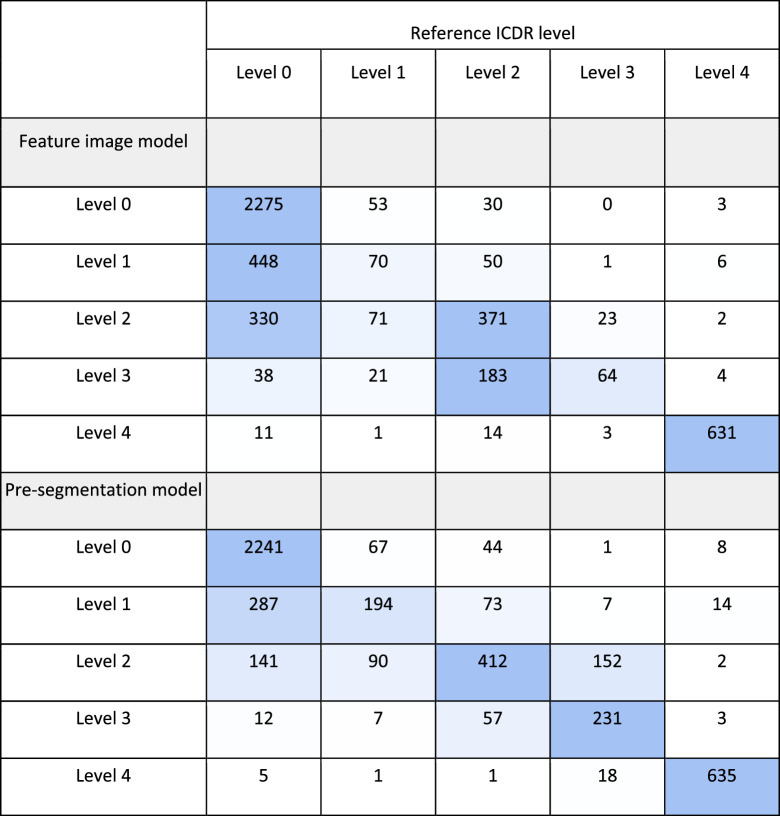

Diagonally colored cells indicate the true positive gradings. The color gradient indicates the number of incorrect gradings for each level relative to the number of correct grades.

**Figure 8.**
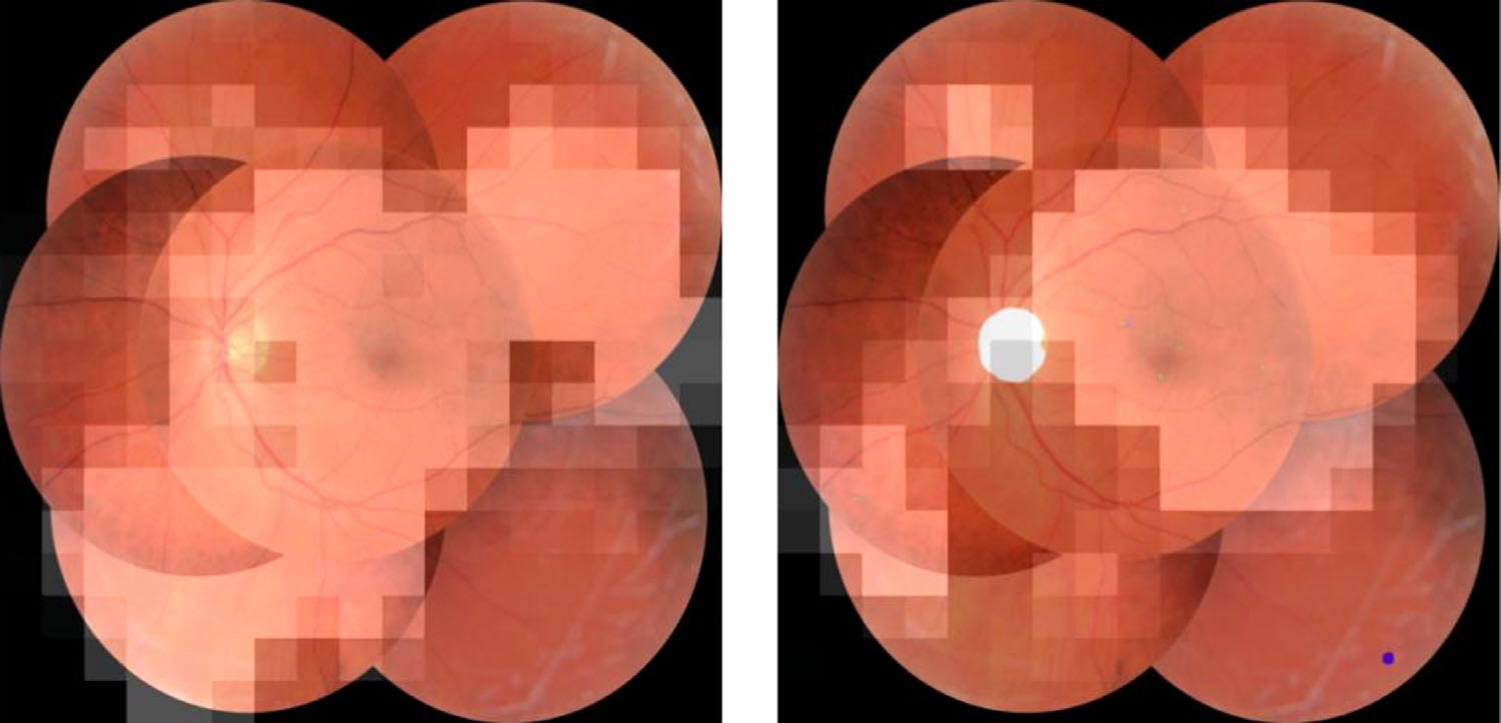
Example image correctly graded as ICDR level 1 by feature image model (*left*) and presegmentation model (*right*) with Grad-CAM heatmaps illustrating regions in the image with high influence on model predictions. In the image on the right, the segmentation model has detected microaneurysms (*green*) as well as a single photocoagulation scar (*purple*).

Figure 8 shows example images correctly graded as ICDR level 1 by both the feature model as well as the model trained on presegmented images. A heatmap created using the gradient weighted class activation map method (Grad-CAM)^33^ is overlaid on the images. This method uses an internal model representation of the image to show regions with high influence on the classification.

**Figure 9.**
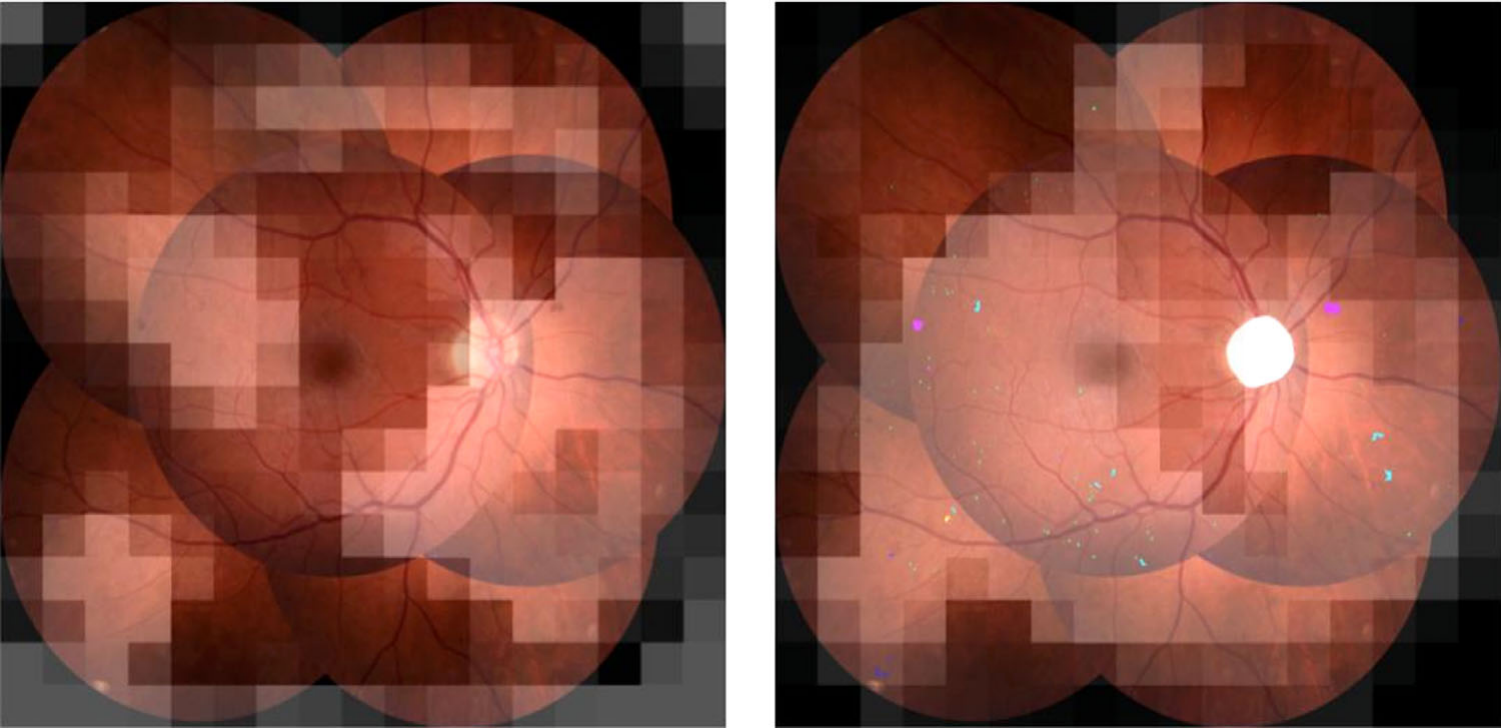
Example image incorrectly graded as ICDR level 2 by feature image model (*left*) but the same image correctly graded as ICDR level 3 by presegmentation model (*right*). Grad-CAM heatmaps illustrate regions with high influence on the prediction. In the image on the *right*, the segmentation model has identified instances of MA (*green*), HEM (*magenta*), IRMA (*cyan*), and NV (*blue*).

Figure 9 gives an example where the feature image model fails to correctly identify the image as ICDR level 3, whereas the model using presegmented images correctly classifies the image.

## Discussion

In this work, we have shown that a deep learning segmentation model can be used for detection of retinal abnormalities associated with DR, achieving similar or better performance for recall and F1 score on several types compared to a retinal expert. The segmentation model can in turn be used in a classification method to improve the grading performance of a classification network for full-scale grading of DR in six-field retinal images. The increased performance was likely due to the segmentation model's ability to detect microvascular abnormalities that otherwise suffer from diminishing feature resolution when images are downscaled prior to development of the classification model.

The ability to recognize and accurately detect microvascular features, such as MA and also HEM, is important as these lesions present in early stages of DR and indicate the risk of progressing to more severe levels of disease.^1^ The segmentation model demonstrated higher recall for detection of individual abnormalities of both types at similar levels of precision compared to a retinal expert. The segmentation model also closely matched the expert in the ability to identify images containing any of these abnormalities, suggesting that segmentation models alone may be used as a tool for identifying patients at risk of progressing to more severe stages of disease.

IRMA and neovascularizations indicate more severe DR. The segmentation model was able to identify more IRMA changes compared to the retinal expert with higher F1 scores, although with lower levels of precision. Because IRMA alone can indicate level 3 DR, the segmentation model may also assist in detecting more severe levels of disease. Compared to the retinal expert, the segmentation model somewhat struggled to accurately detect NV, with both lower recall and precision values. Identifying NV is crucial, as these may result in acute loss of vision due to vitreous hemorrhages. NV was the abnormality type with the fewest examples in the segmentation data, with only 157 instances in the training set. This is significantly less than the number of MA and PC with 11,024 and 7,148 training examples, respectively. Improving performance for this abnormality could then simply be a matter of collecting more data. This process, however, is very cumbersome as it involves annotating individual pixels.

Generally, precision was lower for the segmentation model compared to the expert for both image level detection as well as detection of individual abnormalities. The low model precision could in some cases be attributed to it confusing abnormalities with similar characteristics. This was also the case for the second expert, but was more pronounced for the model. For both the model and expert, MA were often confused with HEM and IRMA, and HEM were likewise confused with IRMA and MA, and for the model in some cases also NV. For the model, IRMA was more often than other abnormalities confused with NV, indicating a similarity between these types of abnormalities. This can perhaps be realized by looking at the example in Figure 4. As seen in the supplementary material, precision increased significantly for both the expert and model when all abnormalities were treated as the same class, that is, when the task was formulated as a binary segmentation/detection problem.

Highly sensitive models can lead to many false positives and this may be problematic. It could be argued that slightly oversensitive models are not necessarily problematic for detection of retinal abnormalities. As it stands, most decisions regarding treatment of DR are handled by humans. Few machines are given full autonomy when it comes to diagnosing DR, and most deep learning models developed for automatic retinal image analysis will therefore operate as clinical decision support tools. As opposed to classification algorithms, segmentation models yield semantically meaningful information directly interpretable by humans, and predictions from overly sensitive models can quickly be verified or ignored. For image level detection, the segmentation model raised a false alarm in 10 out of the 46 test images in the segmentation data set. That is, in 10 images, the model detected abnormalities but none were present according to the expert reference. Of these 10 images, 5 were incorrectly predicted to contain MA, 1 with CWS, 2 with HE, and 2 with PC. Neither HE and CWS alone indicate DR but may be used as indicators of other types of diabetic eye disease, for example, diabetic macular edema. Of the seven images that were incorrectly predicted by the model to contain IRMA, all contained at least MA. Six images also contained HEM, and five of the images in addition contained HE according to the reference. Similarly, 11 images were incorrectly predicted to contain NV, but of those 11 images, 8 of them had been annotated with IRMA, HEM, MA, HE, CWS, HE, and PC by the expert annotator. No images without any abnormalities were incorrectly predicted to contain either IRMA or NV by the model.

Although the image level precision for detecting photocoagulation scar tissue was low, this may not be reason for much concern. In most cases, clinicians will have access to patient health records wherein it is documented whether patients have received prior treatment. As such, this marker is not the most vital for clinical decision support. On the other hand, NV are indicative of DR requiring treatment, and it is therefore problematic that the model had a low recall compared to the expert for image level detection, with lower level of precision as well. As only seven images with NV were present in the test data, the conclusions drawn from these results have to be considered with some degree of uncertainty.

Segmentation and detection of retinal abnormalities can be leveraged for automatic full scale DR disease staging. Presegmentation likely helps minimize the adverse effect of diminishing feature resolution caused by downscaling images prior to development of grading models. The segmentation mask makes it easier for the grading model to recognize relevant features, as these will be more visible in the color-coded segmentation masks. Intuitively, from the point of view of a grading network, recognizing identically colored pixels indicative of specific abnormalities, for example, cyan, magenta, and green for IRMA, HEM, and MA, respectively, is a much more reasonable task compared to the raw pixels values that are affected by pigmentation and image artifacts, such as illumination. This is exemplified in Figure 7, where the feature resolution of microvascular changes in the form of MA are shown in the original resolution image and compared to downsampled images with and without presegmented abnormalities. The problem of reduced feature resolution is also discussed by both Sahlsten et al.^12^ and Krause et al.^13^ In both studies, increased input image resolution during model development led to improved full scale grading performance.

Individual grades on the ICDR scale are, in some cases, defined by specific lesions and microvascular abnormalities. In the case of ICDR level 1 and level 3, MA, HEM, and IRMA may be used as indicators and by including all these abnormalities in the segmentation data set, the classification model was more likely to take these into consideration when leveraging the outputs from the segmentation model. As again illustrated in Figure 7, MA and IRMA are especially sensitive to the adverse effect of downsampling. Looking at the chart in Figure 6, it can be seen how presegmentation of abnormalities leads to improved grading accuracy for these two levels in particular.

The general idea of leveraging segmentation of retinal abnormalities for improved disease staging is analogous to the method by De Fauw et al.^34^ for diagnosis of retinal disease in optical coherence tomography images and also by Ling et al.^35^ for grading DR across multiple levels in retinal images.

As illustrated by the examples in Figures 3, 4, and 5, the segmentation masks created from model predictions were not fully accurate. In some cases, segmentation errors were caused by noise or artifacts in the images, such as underexposure. Hence, it was beneficial to include the raw features as well, rather than relying solely on the segmentation mask when developing the grading model. In some cases, the segmentation model incorrectly detected PC or NV in images that otherwise contained no abnormalities, or with only mild levels of pathology, which could have caused the classification model to incorrectly classify images as level 4 based on the presence of these abnormalities if relying solely on the segmentation mask. We believe that including the raw image features enabled the classification model to reason about the general makeup of an image and take into account the image artifacts that may have caused the segmentation model to fail.

Using the segmentation masks in the grading pipeline also helped to decrease the opaqueness of model predictions by providing semantically meaningful information on what features the model considered when grading images. When comparing the images in Figure 8, it can be seen that the Grad-CAM heatmap from the network trained on presegmented images is more focused on the area where the segmentation model had identified microaneurysms, which are defining of level 1 DR, whereas the corresponding heatmap for the model trained on raw features is more spread out. In the same way, it can be seen in Figure 9 that the model trained on presegmented images was seemingly able to use the IRMA changes detected by the segmentation model to correctly identify the image as representing DR level 3. In comparison, the heatmap from the model trained on raw image features reveals that this network has more or less ignored the regions with IRMA changes, likely causing it to misclassify the image as level 2.

Increased model interpretability is likely going to be a factor in implementing computer assisted diagnostic tools in clinical practice in the future. The proverbial black box nature of convolutional neural networks may serve as a barrier in this regard. Methods such as Grad-CAM aim to resolve this issue by using internal model representations of the image to compute the features most relevant for predictions. Although this method has worked very well for images of more general nature, for example, pictures of animals or everyday objects such vehicles and household items,^33^ it does not yield the same degree of meaningful information when dealing with the high-resolution retinal images used in this study. This is again illustrated by the example in Figure 8, where the heatmaps in both the case of raw feature image and presegmented image are very coarse. Were it not for the presegmented MA, neither image would provide a lot of useful insight into the model's decision making. The shortcomings of the method in the context of medical imaging likely relates to the combination of high-resolution images and more or less microscopic disease markers. In order to get a good indication of important image features, the internal representation is taken from the deep layers of the network where the resolution, that is, height and width of the image, is even smaller than the original input resolution to the network. When the information from this layer is projected back onto the input image, the granularity is decreased, resulting in these types of coarse heatmaps. Thus, the presegmentation approach not only helps to improve grading accuracy, but also significantly increases model interpretability.

When using the segmentation masks in the classification pipeline there was a risk that the grading model would become overly reliant on these and perhaps ignore other features that may be relevant. We attempted to avoid this issue by including most of the known retinal abnormalities in DR in the segmentation data set, including HE and CWS, and not only those defined in the ICDR scale as indicators of different DR disease levels. Currently, disease staging is based on definitions made by human experts. Although these definitions are built on years of cumulative knowledge by many experts, they still may not be perfect in regard to accurately estimating the risk of disease progression or blindness. We imagine that deep learning models may be used for constructing better risk stratification models in the future, and it was therefore a priority to include as much information as possible in the data set to allow models to take this into account.

Making pixel level annotations of abnormalities is an enormously straining and tiresome task, not least in the case of DR, as these are mostly microscopic and hard to discern, even for domain experts. When comparing one expert against another, or a model against an expert as in this study, there is a risk that the results have been influenced by annotators suffering from fatigue. Based on the high level of agreement and consistency demonstrated in our previous study on the agreement between the two experts,^29^ it is our view that it has not affected the results presented here.

In this study, full scale grading of DR in six-field retinal images from Danish patients has been demonstrated for the first time, with results comparable to those demonstrated for two-field retinal images by. Sahlsten et al.^12^ and single field images by Krause et al.^13^ The average per class accuracy for the 5 levels on the ICDR scale was 60.2% and 72.6% in Sahlsten et al.^12^ and Krause et al.,^13^ respectively. Sahlsten et al.^12^ also report a multiclass macro area under the curve value of 0.96. Quadratic weighted Kappa is reported by both Sahlsten et al.^12^ and Krause et al.^13^ with values of 0.91 and 0.84. In comparison, the method described in this study using presegmented images, average per class accuracy was 70.4%, macro area under the curve was 0.92, and quadratic weighted kappa was 0.90.

In this study, the classification models were developed for grading DR across all levels of disease in the ICDR scale. Deep learning models, such as that by Gulshan et al.^5^ that perform binary classification of nonreferable or referable DR, can serve as tools for reducing the strain on healthcare systems by referring only patients with moderate or worse DR to consultations with retinal experts. Automatically grading disease across all levels may hold additional value in regard to reducing healthcare expenditure. Although some countries perform regular screenings of patients regardless of their level of disease, the screening system in Denmark assigns individualized screening intervals based on, among other things, the specific ICDR disease level.^36^ In this setup, the difference in screening interval between level 2 DR and level 3 DR could be as high as 21 months, and, in either case, the patient is not deemed to be in immediate need of medical attention.

Although comparisons are made in this study between the segmentation model and a human expert for detection of retinal abnormalities, this is not the case for full scale grading of DR. At the time of writing, the image grades in the classification data set have been assigned on the basis of electronic health records and the data set has not been subject to further adjudication by retinal experts. The importance of adjudication and expert validation of data sets has been discussed by Gulshan et al.^5^ and Krause et al.^13^ and this is something that will need to be addressed in the future. Comparisons between a CNN and retinal specialists for full scale grading of DR is made by Krause et al.,^13^ where the quadratic weighted kappa values for human experts ranged from 0.80 to 0.91. As such, the method presented here could be argued to perform on the level of human experts.

The results presented in this study suggest that segmentation models can serve as an additional tool for clinical decision support and automated grading of DR. By the virtue of the unique segmentation data set presented here, along with adjudication of classification data, it should be possible to develop more effective models in the future.

## Supplementary Material

Supplement 1
