# Supplementary material for: Automatic Detection of Abnormalities and Grading of Diabetic Retinopathy in 6-Field Retinal Images: Integration of Segmentation Into Classification
Source: Transl Vis Sci Technol. 2022 Jun 22;11(6):19. doi: 10.1167/tvst.11.6.19 (PMC9233290; doi:10.1167/tvst.11.6.19)
Supplement: Supplement 1 [file tvst-11-6-19_s001.pdf]

		MA	HEM	CWS	HE	PC	IRMA	NV	Adjusted Mean	Original mean
Model	Precision	0.75	0.84	0.57	0.67	0.84	0.62	0.64	0.70	0.52
Expert 2	Precision	0.79	0.88	0.83	0.71	0.91	0.68	0.88	0.81	0.66

Supplementary Table 1 Precision values of the model and second expert for detecting any abnormality. I.e, each detected abnormality is counted as true positive if it overlaps with any abnormality in the reference image. Adjusted mean refers to the mean precision when all abnormalities are treated as equal. Original mean indicates the value when abnormality types are differentiated. MA = microaneurysms, HEM = hemorrhages, CWS = cotton wool spots, HE = hard exudates, PC = photocoagulation scars, IRMA = intraretinal microvascular abnormalities, NV = neovascularizations.
